# Male and Female Sensitivity to Alcohol-Induced Brain Damage

**Published:** 2003

**Authors:** Daniel W. Hommer

**Affiliations:** Daniel W. Hommer, M.D., is chief of the Section of Brain Electrophysiology and Imaging, Laboratory of Clinical Studies, Division of Intramural Clinical and Biological Research, at the National Institute on Alcohol Abuse and Alcoholism, Bethesda, Maryland

**Keywords:** AODR (alcohol and other drug related) structural brain damage, AOD sensitivity, gender differences, AODR biological markers, cerebrospinal fluid, hippocampus, corpus callosum, cerebral cortex, brain imaging

## Abstract

Women are more vulnerable than men to many of the medical consequences of alcohol use. Although research has shown that male alcoholics generally have smaller brain volumes than nonalcoholic males, the few studies that have compared brain structure in alcoholic men and women have had mixed results. To adequately compare brain damage between alcoholic women and men, it is necessary to control for age and to have separate control groups of nonalcoholic men and women. Although the majority of studies suggest that women are more vulnerable to alcohol-induced brain damage than men, the evidence remains inconclusive.

Men and women are affected differently by many diseases, including alcohol-related conditions. For at least a quarter century researchers have recognized that many of the medical consequences of excessive alcohol consumption develop more rapidly among women than among men (Ashley et al. 1977). For example, alcoholic women develop cirrhosis (Loft et al. 1987), alcohol-induced weakening of the heart muscle (i.e., cardiomyopathy) (Fernandez-Sola et al. 1997), and nerve damage in the body’s extremities (i.e., peripheral neuropathy) (Ammendola et al. 2000) after fewer years of heavy drinking than alcoholic men. Studies comparing men’s and women’s sensitivity to alcohol-induced brain damage, however, have yielded inconsistent results. This article reviews the research and the factors other than alcoholism that can affect gender-based comparisons of brain structure.

## Male and Female Vulnerability to Alcohol-Induced Brain Damage

As reviewed in this issue of *Alcohol Research & Health* (see the article by Rosenbloom and colleagues), many studies have found small but usually statistically significant differences in the brain volumes of male alcoholics compared with those of nonalcoholics. In contrast, few studies have directly compared brain structure between alcoholic men and alcoholic women. Two early computerized tomography studies (Jacobson 1986; Mann et al. 1992) compared brain shrinkage, a common marker of brain damage, in alcoholic men and women by measuring the increase in the fluid surrounding the brain (i.e., cerebrospinal fluid [CSF]), which is an indication of the size of the lateral ventricle (a CSF-filled cavity inside the brain that increases in size as the brain shrinks).

Both studies reported that male and female alcoholics had significantly larger amounts of intracranial CSF than control subjects did, indicating greater brain shrinkage among alcoholics of both genders; alcoholic women also reported about half as many years of excessive drinking as the alcoholic men. In addition to this evidence for excessive brain shrinkage among alcoholic women, there is also evidence that the degree of cognitive dysfunction in alcoholic women is similar to that in alcoholic men despite fewer years of heavy drinking on the part of the women (Nixon et al. 1995). These results suggested that the central nervous system (CNS) in women, like other organ systems, is more vulnerable to alcohol-induced damage than the CNS in men.

Subsequent studies have not universally confirmed women’s greater vulnerability to alcohol-induced brain damage. For example, Kroft and colleagues (1991), using magnetic resonance imaging (MRI), failed to detect that the fluid-filled chambers in the brain (i.e., cerebral ventricles) were larger in alcoholic women than nonalcoholic women, although other researchers have found MRI evidence for ventricular enlargement among alcoholic men (Pfefferbaum et al. 1993). Two recent reports that appeared side by side in the *American Journal of Psychiatry* contradicted each other on the question of gender-related vulnerability to brain shrinkage in alcoholism (Hommer et al. 2001; Pfefferbaum et al. 2001).

It would seem that whether alcoholic women experience greater brain damage than alcoholic men could be determined by comparing the sizes of the brains of alcoholic men and women with the brain sizes of nonalcoholics of each gender. However, details such as how different investigators measure brain size and damage or how they select alcoholic subjects may make a big difference in the results of each study. Because relatively few studies have compared alcoholic men and women, it is possible to review the measurement methods and population characteristics used in each study to determine if these factors explain the inconsistent results. Before examining these studies on alcoholism, however, it is useful to describe factors independent of alcoholism, such as age (Gur et al. 2002; Xu et al. 2000), that affect gender differences in brain structure.

### Factors Affecting Gender-Based Differences in Brain Structure

**Figure 1:**
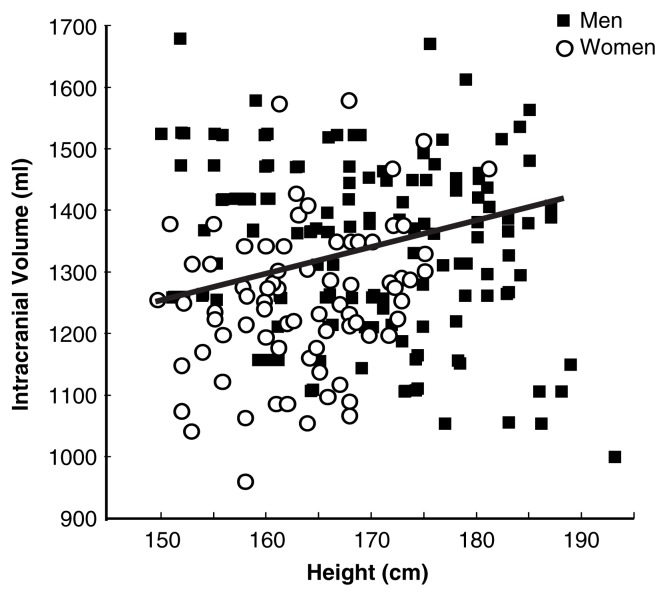
Intracranial volume (a measure of the maximum size to which the brain grows) increases as a function of height among healthy nonalcoholic men and women. Most of the difference in intracranial volume between healthy nonalcoholic women and men is explained by height. SOURCE: Subset of the data (nonalcoholics only) reported in Hommer et al. 2001.

**Figure 2:**
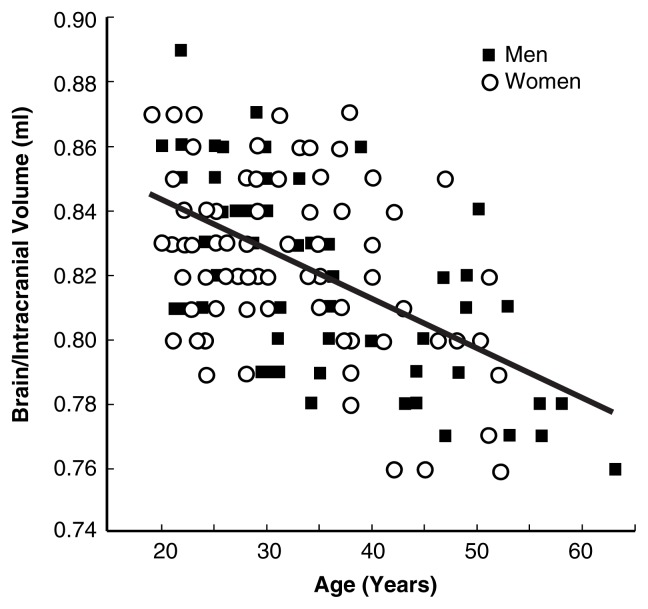
The ratio of brain to intracranial volume (a measure of the maximum size to which the brain grows) decreases as a function of age, among healthy nonalcoholic men and women. The brain shrinks as we age. SOURCE: Subset of the data (nonalcoholics only) reported in Hommer et al. 2001.

**Figure 3:**
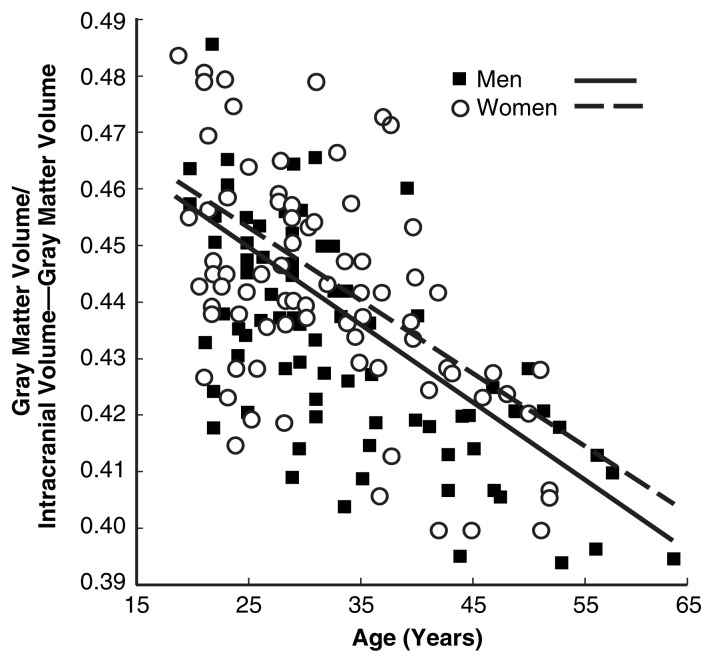
The proportion of intracranial volume occupied by gray matter decreases as healthy nonalcoholic men and women age, but, at any age, women have proportionally more gray matter than men. SOURCE: Subset of the data (nonalcoholics only) reported in Hommer et al. 2001.

To illustrate the factors that influence brain size, figures 1, 2, and 3 compare the brains of healthy nonalcoholic men and women. The data shown were collected using full volumetric MRI scans that were automatically segmented into gray matter, white matter, and CSF (Momenan et al. 1997). Gray matter is the tissue of the nervous system that appears grayish because of the relatively high proportion of nerve cell bodies it contains. White matter is made up of fibers that extend from the nerve cell bodies and carry information between them.

Women have smaller bodies than men, and this difference in body size extends to the head, skull, and brain. As shown in figure 1, most of the difference in intracranial volume (a measure of the maximum size to which the brain grows) between healthy nonalcoholic women and men is explained by height. However, several studies have shown that even when height is taken into account, women still have smaller brains than men (Breedlove 1994; Kretschmann et al. 1979; Swaab and Hofman 1984).

In addition to height, age is another factor influencing brain size. The amount that the brain shrinks with age can be measured by examining the ratio of brain volume to intracranial volume. In this way, brain shrinkage can be measured independent of the initial size of the brain. Among both men and women the ratio of brain volume to intracranial volume decreases with age, as shown in figure 2. Several researchers have reported that the rate of brain shrinkage during aging may be slightly higher among men (Gur et al. 2002; Xu et al. 2000).

Some investigators have found that women’s proportion of gray matter appears to be slightly, but significantly, greater than men’s (Gur et al. 1999). Other researchers have suggested that this difference is secondary to overall brain size and not specifically related to gender (Luders et al. 2002). Figure 3 shows that, among healthy nonalcoholic control subjects, women have proportionally more gray matter than men; it also appears that the proportion of the inside of the skull occupied by gray matter decreases with aging similarly in men and women. However, analyses of gray-matter volume were conducted to remove the influence of overall brain size, the differences in gray matter between the genders were no longer significant. This suggests that the greater proportion of gray matter observed among women is caused by differences in brain size between men and women and not by gender itself.

The results summarized in the three figures indicate that to adequately compare brain damage between alcoholic women and men, researchers should control for age and have separate control groups of nonalcoholic women and men. Separate control groups are needed, for example, because differences in height account for most of the difference in brain size between men and women. Controlling for the amount of lifetime alcohol consumption also is necessary because several studies have shown that alcohol consumption, independent of age, predicts brain shrinkage (Bjork et al. in press; Pfefferbaum et al. 1998).

### Studies of Brain Size in Alcoholic Men and Women

Only one study has compared the volumes of specific brain structures of alcoholic and nonalcoholic men and women (Agartz et al. 2003). This study measured the volume of the hippocampus, a structure critical for memory function, and found that alcoholic women had smaller right and left hippocampi than nonalcoholic women; alcoholic men only had smaller right hippocampi in comparison with nonalcoholic men. The alcoholic women reported fewer years of heavy drinking, a later age of onset, and lower estimated lifetime alcohol consumption compared with the alcoholic men. DeBellis and colleagues (2000) also reported smaller hippocampal volumes (both right and left) in a small group of adolescent alcoholics compared with their nonalcoholic peers. The authors did not report the results by gender.

Two studies examined the size of the corpus callosum, a large fiber bundle connecting the two cerebral hemispheres, which is essential for communication between the hemispheres. Reduction in the size of the corpus callosum may be a marker for damage to long white-matter tracts connecting the two hemispheres, and thus an indicator of the overall health of the brain’s white matter. One study showed that after controlling for brain size, alcoholic women had significantly smaller corpus callosum areas than both nonalcoholic women and alcoholic men (Hommer et al. 1996). However, other research has failed to find significant differences between alcoholic and nonalcoholic women in corpus callosum area (Pfefferbaum et al. 2002). The methods used to measure corpus callosum size were similar in these studies, and the severity of alcoholism in the two samples did not differ. The reason for the inconsistent results is not clear. Subsequent studies, however, using more sensitive measures to evaluate white-matter microstructure (i.e., diffusion tensor imaging [DTI], described in the article in this issue by Rosenbloom and colleagues) have reported evidence for brain damage among alcoholic women when compared with female control subjects matched for brain size (Pfefferbaum and Sullivan 2002). It is possible that the white-matter damage in female alcoholics may be better detected using DTI than conventional MRI techniques.

Only two studies have compared total cerebral volume among alcoholic and nonalcoholic men and women (Agartz et al. 2003; Hommer et al. 2001). Both studies used high-resolution, full-volumetric MRI scanning techniques and found strong evidence for greater brain shrinkage among alcoholic women compared with alcoholic men, even though the alcoholic women started heavy drinking later in life and had consumed less alcohol in their lifetimes. (However, in both studies, alcoholic women drank as much as or more than alcoholic men in the 6 months immediately preceding the scan, suggesting that brain volume may be more influenced by recent alcohol consumption than by earlier alcohol use.)

In another approach, Pfefferbaum and colleagues (2001) used an MRI method that sampled a block of tissue made up of a set of axial slices (i.e., tissue sliced along the horizontal plane) comprising approximately 25 percent of total brain volume. This study found less brain shrinkage among alcoholic women than men.

**Table:** Studies of Gender Differences in Alcohol-Induced Brain Damage

Study	Findings	Imaging Method
Jacobson 1986	Male and female alcoholics had significantly more cerebrospinal fluid (CSF) than did control subjects.	computerized tomography
Mann et al. 1992	Alcoholic women had shorter histories of heavy drinking and less average daily consumption than did alcoholic men.	computerized tomography
Kroft et al. 1991	Cerebral ventricles in alcoholic women were not larger than those in nonalcoholic women.	MRI
Pfefferbaum et al. 1993	Cerebral ventricles in alcoholic men were larger than those in nonalcoholic men.	MRI
Hommer et al. 2001	Strong evidence indicated greater brain shrinkage among alcoholic women compared with alcoholic men, even though the alcoholic women started heavy drinking later in life and had consumed less alcohol in their lifetimes.	full volumetric MRI scans that automatically segmented gray/white matter and CSF
Pfefferbaum et al. 2001	Less brain shrinkage was found among alcoholic women than among alcoholic men (few subjects had a history of hospitalization).	MRI that sampled axial slices of tissue (25 percent of total brain volume)
Agartz et al. 2003	Greater global brain shrinkage and smaller hippocampi were found among alcoholic women than among alcoholic men (although women had a later onset of heavy drinking).	full volumetric MRI scans that automatically segmented gray/white matter and CSF; hippocampi outlined by hand
Hommer et al. 1996	When brain size was controlled, alcoholic women had significantly smaller corpus callosums than did nonalcoholic women or alcoholic men.	MRI; corpus callosum outlined by hand
Pfefferbaum et al. 2002	No significant differences were found between alcoholic and nonalcoholic women in corpus callosum area.	MRI; corpus callosum outlined by hand
Pfefferbaum and Sullivan 2002	An evaluation of white-matter microstructure found evidence for brain damage in alcoholic women but not female control subjects.	diffusion tensor imaging
Schweinsburg et al. 2003	Both male and female alcoholics had reductions in frontal lobe white-matter N-acetylaspartate (a marker for neuronal activity), but only alcoholic females had reduction in gray-matter N-acetylaspartate.	magnetic resonance spectroscopy (MRS) to measure concentrations of N-acetylaspartate.

The inconsistency between the results obtained by Pfefferbaum and colleagues and the results from the previously described studies (Agartz et al. 2003; Hommer et al. 2001) does not appear to be accounted for by differences in lifetime alcohol consumption between the samples of alcoholic women, although few of the women in the Pfefferbaum study had a history of hospitalization for alcoholism, and all of the women in the other two studies had been hospitalized at some point. It is possible that differences in imaging methods contributed to the different results, because Pfefferbaum’s study measured only a portion of the cerebrum, and the other studies measured the volume of the entire cerebrum.

In addition to the studies of brain structure reviewed above, one study using magnetic resonance spectroscopy (MRS) to compare male and female alcoholics (Schweinsburg et al. 2003) found that, in frontal lobe gray matter but not frontal lobe white matter, alcoholic women had a significantly greater deficit in concentrations of N-acetyl-aspartate (a marker for neuronal integrity) than alcoholic men. These MRS findings are consistent with the report by Hommer and colleagues (2001) that gray-matter damage distinguishes alcoholic women and men to a greater extent than white-matter damage.

## Conclusion

Compared with alcoholic men, alcoholic women have received little research attention. This is particularly unfortunate because there is good evidence that many of the behavioral aspects of alcoholism progress more rapidly among women than among men. It is important to determine what role, if any, alcoholism-related brain damage plays in the progression of alcoholism. Although most of the recent studies suggest that women are more vulnerable to alcohol-induced brain damage than men, it would be premature to state this conclusively. Nearly all the recent studies of gender differences in this area have been conducted by only two research groups, one of which (Hommer and colleagues) has consistently found evidence for greater brain damage among alcoholic women, whereas the other group (Pfefferbaum and colleagues) has not. The field of gender studies in alcoholism needs more investigators to join in this work in order to resolve the conflicting results that currently characterize this field.

## References

[b1-181-185] Agartz I, Shoaf S, Rawlings RR (2003). CSF monoamine metabolites and MRI brain volumes in alcohol dependence. Psychiatry Research: Neuroimaging.

[b2-181-185] Ammendola A, Gemini D, Iannaccone S (2000). Gender and peripheral neuropathy in chronic alcoholism: A clinical-electroneurographic study. Alcohol and Alcoholism.

[b3-181-185] Ashley MJ, Olin JS, le Riche WH (1977). Morbidity in alcoholics. Evidence for accelerated development of physical disease in women. Archives of Internal Medicine.

[b4-181-185] Bjork J, Grant S, Hommer D Cross-sectional volumetric analysis of brain atrophy in alcoholics: Effects of drinking history and comorbid substance use disorder. American Journal of Psychiatry.

[b5-181-185] Breedlove SM (1994). Sexual differentiation of the human nervous system. Annual Review of Psychology.

[b6-181-185] DeBellis MD, Clark DB, Beers SR (2000). Hippocampal volume in adolescent-onset alcohol use disorders. American Journal of Psychiatry.

[b7-181-185] Fernandez-Sola J, Estruch R, Nicolas JM (1997). Comparison of alcoholic cardiomyopathy in women versus men. American Journal of Cardiology.

[b8-181-185] Gur RC, Turetsky BI, Matsui M (1999). Sex differences in brain gray and white matter in healthy young adults: Correlations with cognitive performance. Journal of Neuroscience.

[b9-181-185] Gur RC, Gunning-Dixon FM, Turetsky BI (2002). Brain region and sex differences in age association with brain volume: A quantitative MRI study of healthy young adults. American Journal of Geriatric Psychiatry.

[b10-181-185] Hommer D, Momenan R, Rawlings R (1996). Decreased corpus callosum size among alcoholic women. Archives of Neurology.

[b11-181-185] Hommer DW, Momenan R, Kaiser E, Rawlings RR (2001). Evidence for a gender-related effect of alcoholism on brain volumes. American Journal of Psychiatry.

[b12-181-185] Jacobson R (1986). The contributions of sex and drinking history to the CT brain scan changes in alcoholics. Psychological Medicine.

[b13-181-185] Kretschmann HJ, Schleicher A, Wingert F (1979). Human brain growth in the 19th and 20th century. Journal of Neurological Science.

[b14-181-185] Kroft CL, Gescuk B, Woods BT (1991). Brain ventricular size in female alcoholics: An MRI study. Alcohol.

[b15-181-185] Loft S, Olesen KL, Dossing M (1987). Increased susceptibility to liver disease in relation to alcohol consumption in women. Scandinavian Journal of Gastroenterology.

[b16-181-185] Luders E, Steinmetz H, Jancke LCA (2002). Brain size and grey matter volume in the healthy human brain. Neuroreport.

[b17-181-185] Mann K, Batra A, Gunthner A, Schroth G (1992). Do women develop alcoholic brain damage more readily than men?. Alcoholism: Clinical and Experimental Research.

[b18-181-185] Momenan R, Hommer D, Rawlings R (1997). Intensity adaptive segmentation of single echo T_1_-weighted magnetic resonance images. Human Brain Mapping.

[b19-181-185] Nixon S, Tivis R, Parsons O (1995). Behavioral dysfunction and cognitive efficiency in male and female alcoholics. Alcoholism: Clinical and Experimental Research.

[b20-181-185] Pfefferbaum A, Sullivan EV (2002). Microstructural but not macrostructural disruption of white matter in women with chronic alcoholism. NeuroImage.

[b21-181-185] Pfefferbaum A, Sullivan EV, Rosenbloom MJ (1993). Increase in brain cerebrospinal fluid volume is greater in older than in younger alcoholic patients: A replication study and CT/MRI comparison. Psychiatry Research.

[b22-181-185] Pfefferbaum A, Sullivan EV, Rosenbloom MJ (1998). A controlled study of cortical gray matter and ventricular changes in alcoholic men over a 5-year interval. Archives of General Psychiatry.

[b23-181-185] Pfefferbaum A, Rosenbloom M, Deshmukh A, Sullivan E (2001). Sex differences in the effects of alcohol on brain structure. American Journal of Psychiatry.

[b24-181-185] Pfefferbaum A, Rosenbloom M, Serventi KL, Sullivan EV (2002). Corpus callosum, pons, and cortical white matter in alcoholic women. Alcoholism: Clinical and Experimental Research.

[b25-181-185] Schweinsburg B, Alhassoon O, Taylor M (2003). Effects of alcoholism and gender on brain metabolism. American Journal of Psychiatry.

[b26-181-185] Swaab DF, Hofman MA (1984). Sexual differentiation of the human brain. A historical perspective. Progress in Brain Research.

[b27-181-185] Xu J, Kobayashi S, Yamaguchi S (2000). Gender effects on age-related changes in brain structure. American Journal of Neuroradiology.

